# Standardized evaluation of Zika nucleic acid tests used in clinical settings and blood screening

**DOI:** 10.1371/journal.pntd.0011157

**Published:** 2023-03-17

**Authors:** Mars Stone, Sonia Bakkour, Eduard Grebe, Devy M. Emperador, Camille Escadafal, Xutao Deng, Honey Dave, Cassandra Kelly-Cirino, Eve Lackritz, Diana P. Rojas, Graham Simmons, Ingrid B. Rabe, Michael P. Busch

**Affiliations:** 1 Vitalant Research Institute, San Francisco, California, United States of America; 2 Department of Laboratory Medicine, University of California San Francisco, San Francisco, California, United States of America; 3 Pandemic Threats Program, FIND, Geneva, Switzerland; 4 Elizabeth Glaser Pediatric AIDS Foundation, Geneva, Switzerland; 5 World Health Organization, Geneva, Switzerland; The University of Kansas, UNITED STATES

## Abstract

Early detection of Zika virus (ZIKV) transmission within geographic regions informs implementation of community mitigation measures such as vector reduction strategies, travel advisories, enhanced surveillance among pregnant women, and possible implementation of blood and organ donor screening or deferral. Standardized, comparative assessments of ZIKV assay and testing lab performance are important to develop optimal approaches to ZIKV diagnostic testing and surveillance. We conducted an expanded blinded panel study to characterize and compare the analytical performance of fifteen diagnostic and blood screening ZIKV NAT assays, including detection among single- and multiplex assays detecting ZIKV, dengue virus (DENV) and chikungunya virus (CHIKV). A 300 member blinded panel was constructed, consisting of 11 serial half-log dilutions ranging from ~10^4^ to 10^−1^ genome equivalents/mL in 25 replicates each of the Tahitian Asian ZIKV isolate in ZIKV-negative human serum. Additionally, clinical samples from individuals with DENV-like syndrome or suspected ZIKV infection in Brazil were evaluated. The majority of assays demonstrated good specificity. Analytical sensitivities varied 1–2 logs, with a substantially higher limit of detection (LOD) in one outlier. Similar analytical sensitivity for ZIKV RNA detection in singleplex and multiplex assays of the Grifols and ThermoFisher tests were observed. Coefficient of Assay Efficiency (CE), calculated to characterize assays’ RNA extraction and amplification efficiency, ranged from 0.13 for the Certest VIASURE multiplex and 0.75 for the Grifols multiplex assays. In general, assays using transcription mediated amplification (TMA) technology had greater CE compared to assays using conventional PCR technology. Donor screening NAT assays were significantly more sensitive than diagnostic RT-qPCR assays, primarily attributable to higher sample input volumes. However, ideal assays to maximize sensitivity and throughput may not be a viable option in all contexts, with other factors such as cost, instrumentation, and regulatory approval status influencing assay availability and selection, particularly in resource constrained settings.

## Author summary

Having standardized, comparative studies of Zika virus (ZIKV) tests are important for implementing optimal diagnostic testing and disease surveillance. Detection of ZIKV nucleic acid is ideal for confirming infection; however, knowing the sensitivity and specificity of a test is needed for selecting the right test in a given laboratory. We looked at the analytical performance of 15 diagnostic and blood screening ZIKV molecular tests, including tests that detect ZIKV only and tests that detect ZIKV plus other arboviruses. We found donor screening NAT assays were significantly more sensitive than diagnostic RT-qPCR assays, mostly due to the higher sample input volumes. We also found no difference in analytical sensitivity between tests that only detected ZIKV compared to tests that detected ZIKV and related viruses. The ideal molecular test will depend on the testing context, with other factors such as cost, instrumentation, and regulatory approval status influencing assay availability and selection.

## Introduction

Zika virus (ZIKV) spread rapidly throughout most of Latin America during 2015 and early 2016, resulting in severe neurological consequences in fetuses and adults. Although ZIKV is mostly transmitted through the bite of *Aedes* mosquitoes, [1] other routes of transmission have been identified. [1–8] Of particular public health concern, vertical transmission during pregnancy can cause Congenital ZIKV Syndrome in a proportion of cases (CZS) [9–13] and this can occur whether maternal ZIKV infection is symptomatic or asymptomatic. In addition, ZIKV has the potential for transfusion transmission (TT) with a high proportion of documented ZIKV RNAemia in donors during the 2013–2014 French Polynesia outbreak and the 2016 outbreak in the Americas [14,15] and several cases of probable transfusion transmission reported in Brazil. ZIKV ribonucleic acid (RNA) and infectious ZIKV has been found in whole blood, [15], urine, [3] breast milk, [16] saliva and semen [17,18].

Timely public health interventions and appropriate clinical management depend on accurate detection of acute ZIKV infections. Early detection of ZIKV transmission activity within geographic regions serve to guide implementation of community mitigation measures such as vector reduction strategies, travel advisories, enhanced surveillance among pregnant women, and possible implementation of blood and organ donor screening, donor deferral, or pathogen reduction techniques in blood products.

Most ZIKV infections are asymptomatic or mildly symptomatic with non-specific symptoms, limiting the ability of presenting clinical cases to assess ZIKV infection rates/incidence. Testing for the presence of ZIKV RNA is a more accurate and specific means of diagnosing ZIKV infections during outbreaks and, given the risk for transfusion transmission, may be implemented for screening the blood supply. ZIKV antibody testing is impacted by extensive antibody cross-reactivity between ZIKV and dengue viruses (DENV) and other antigenically similar flaviviruses and detects seroreactivity after acute viremia is resolved and thus more limited in value. For this reason, the US Food and Drug Administration (FDA), [19] European Center for Disease Control (ECDC) [20] and the World Health Organization (WHO) [21] have recommended nucleic acid technology (NAT) tests for detection of acute ZIKV infection, and particularly for blood donor screening [22,23].

When the first cases of ZIKV infection in the Americas were reported in 2015 there were no commercial assays available to test for the presence of ZIKV RNA or antibodies. Government, academic laboratories and industry responded rapidly to this emerging threat to develop sensitive and specific assays, including high throughput assays essential to ensure a safe and adequate blood supply should donor screening be recommended. However, NAT performance can vary widely depending on assay characteristics, thus comparative assessments of assay and testing lab performance relative to a reference standard is critical.

Although ZIKV transmission in the Americas has greatly declined since late 2017, the inter-epidemic period was identified as a critical time to advance research, development, and availability of ZIKV diagnostics to continue public health programs in endemic areas and prepare for future outbreaks. Standardized, comparative assessments of performance of different ZIKV diagnostic assays and testing labs are important for clinicians, public health organizations, and emergency response planners to develop optimal approaches to ZIKV diagnostic testing and surveillance.

At the beginning of the ZIKV epidemic, with the impending mandate for ZIKV screening of blood donations in Puerto Rico and US states, there was an urgent need to evaluate the performance of newly developed, donor NAT screening assays to be implemented under IND authorization, relative to other diagnostic ZIKV assays. We executed a study to compare the performance of blood screening assays demonstrating their excellent sensitivities and appropriateness for use, but that study was not powered for significant precision and did not include many ZIKV RNA assays that were developed for diagnostic use. [24]

We therefore executed an expanded blinded panel study to robustly characterize and compare the analytical performance of diagnostic and blood screening NAT assays for ZIKV RNA using well-characterized and precisely quantified viral stocks to generate serially diluted virus panels. The panels were sufficiently powered to establish analytical sensitivity and precise estimation of 50% and 95% limits of detection (LOD_50_ and LOD_95_) of molecular assays for ZIKV, as well as multiplexed assays for ZIKV, DENV, and CHIKV virus [15].

Subsequent to initial execution of the expanded blinded panel study, a number of other ZIKV NAT assays received approval from regulatory agencies. The WHO ZIKV Task Force sought to advance global capacity for ZIKV preparedness and response through support of FIND, the global alliance for diagnostics, to advance diagnostics for emerging global health threats, including ZIKV and other arboviral diseases. The scope of work for the ZIKV Diagnostics Evaluation Project encompassed a standardized evaluation of currently available immunologic and molecular diagnostic tests for ZIKV infection, and the molecular evaluation built off our previous work to evaluate performance of blood screening and diagnostic assays. The results were compiled from both phases and compared across testing labs to evaluate the relative sensitivities of molecular assays currently used in various settings including clinical diagnosis, research and blood donor screening.

## Methods

### Ethics statement

This work was approved by the University of Sao Paulo Medical School Ethical Review Board (CAAE number 89342418.1.0000.0065). Since samples were anonymized prior to testing and participants underwent no additional procedures, individual informed consent was deemed unnecessary for virus detection.

### Panel development

A 300-member blinded ZIKV analytical performance panel was constructed consisting of 11 serial half-log dilutions in 25 replicates each of the Tahitian Asian ZIKV isolate (which was chosen as the WHO international standard) with initial concentrations of ZIKV RNA quantified by RT-qPCR [25]. Serial dilutions were prepared using defibrinated ZIKV-negative human serum (Gemini Biosciences) resulting in panels with ZIKV viral loads (VLs) ranging from ~10^4^ to 10^−1^ genome equivalents/mL. The dilutions were performed in a single procedure at VRI in order to generate a sufficient number of 1mL aliquots of each dilution to generate identical frozen panels with a total of 25 uniquely labelled vials for each member panel to perform 25 replicate runs of the panel; the panel also included 25 replicate negative control diluent samples. The 300-member panels with unique identifiers on each tube were stored at -80°C and shipped on dry ice until testing. Panels were sent to manufacturers, research and commercial laboratories experienced in performing the assays they were evaluating, in **Table 1**. Results were reported to VRI for unblinding and analysis of % reactivity at each dilution and probit regression analysis to estimate LOD_50_ and LOD_95_.

**Table 1:** Summary of key characteristics, including extraction methods, input sample volumes, cutoffs values, and instruments, of the 15 assays performed at the 10 laboratories that participated in this study.

Manufacturer	Assay	Evaluation Phase	Use Type	Plasma extraction volume	Extraction method	Elution Volume	RNA input volume	Derived plasma input*	Instrument
**Roche**	**Roche cobas Zika**	VRI Phase 1	Donor Screening	850ul	magnetic glass particles	Proprietary	Proprietary	~500ul	cobas 6800/8800 Systems
**Grifols**	**Procleix TMA**	VRI Phase 1	Donor Screening	500ul	magnetic bead target capture	N/A	500ul	500ul	Procleix Panther
	**Triplex**	VRI Phase 1	RUO	500ul	magnetic bead target capture	N/A	500ul	500ul	Procleix Panther
**CDC**	**CDC Singleplex HI**	VRI Phase 1	Diagnostic	1000ul	MagNApure 96	100ul	10ul	100ul	ABI7500Dx
	**CDC Trioplex HI**	VRI Phase 1	Diagnostic	1000ul	MagNApure 96	100ul	10ul	100ul	ABI7500Dx
	**CDC Trioplex LI**	VRI Phase 1	Diagnostic	200ul	MagNA Pure 96	100ul	10ul	20ul	ABI7500Dx
**Abbott**	**Abbott RealTime PCR Zika**	VRI Phase 1	Diagnostic	500ul	magnetic bead target capture	Proprietary	40ul	~500ul	Abbott m2000rt
**VRI**	**IND NAT confirmatory**	VRI Phase 1	RUO	140ul	QIAamp Viral RNA Mini Kit	60ul	22.7ul in duplicate	106ul	LightCycler 480 real-time PCR
**Altona**	**RealStar Zika Virus RT-PCR**	VRI Phase 1	Diagnostic	140ul	NucleoSpin 96	50ul	10ul	28ul	Microlab STARlet and CFX96 (Bio-Rad)
**Bioneer**	**AccuPower ZIKV (DENV, CHIKV) Multiplex Real-Time RT-PCR Kit**	FIND/WHO Phase II	Diagnostic	400ul	ExiPrep Instrument	80ul	50ul	125ul	Exicycler96 Instrument
**EUROIMMUN AG**	**EUROArray ArboCDZ**	FIND/WHO Phase II	Diagnostic	200ul	QIAamp Viral RNA Mini Kit	50ul	5ul	20ul	Applied Biosystems 2720 Thermal Cycler
**Thermo Fisher Scientific**	**TaqMan Zika Virus Kit, Triplex**	FIND/WHO Phase II	Diagnostic	300uL	MagMAX Pathogen RNA/DNA Kit	50ul	25ul	150ul	QuantStudio Dx Real-Time PCR Instrument
	**TaqPath Zika Virus Kit**	FIND/WHO Phase II	RUO	300uL	MagMAX Pathogen RNA/DNA Kit	50ul	25ul	150ul	QuantStudio Dx Real-Time PCR Instrument
**Certest Biotec**	**VIASURE Zika, Dengue & Chikungunya Real Time PCR Detection Kit**	FIND/WHO Phase II	Diagnostic	400ul	Qiamp Viral RNA Mini kit (QIAGEN)	50ul	5ul	40ul	CFX96 (Bio-Rad)
	**VIASURE Zika Virus Real Time PCR Detection Kit**	FIND/WHO Phase II	Diagnostic	400ul	Qiamp Viral RNA Mini kit (QIAGEN)	50ul	5ul	40ul	CFX96 (Bio-Rad)

* A derived parameter to represent the amount of plasma subjected to amplification

### Panel calibration and quantification

The concentration of viral genome equivalents of the French Polynesian ZIKV isolate was calculated from serial dilutions and real-time RT-qPCR data using a computational method based on maximum-likelihood estimation (MLE) for viral genome equivalents number estimation [26]. The ZIKV RNA end-point was first determined based on 10-fold dilutions of the viral stock in plasma. Subsequently, eight 3-fold dilutions around the end-point were prepared by diluting in ZIKV-negative plasma. Twenty replicate RNA extractions were performed for each of the two highest and two lowest dilutions, whereas 40 replicate RNA extractions were performed for each of the four intermediate dilutions. RNA was reverse transcribed and amplified in quadruplicate wells using two-step real-time RT-PCR, and the number of positive replicates was recorded for each dilution. MLE was used to combine the Poisson distribution estimators for all dilutions and generate a viral genome equivalents number estimate that optimally fit the overall serial dilution data.

An aliquot of the highest concentration in the ZIKV NAT panel was quantified using a confirmatory RT-qPCR assay developed by Vitalant Research Institute [25]. Viral load was extrapolated from a standard curve using four 10-fold serial dilutions of the First International Standard (SI) for ZIKV virus NAT assays (PEI: 11468/16) [27–29]. The viral load from triplicate assay runs (performed by three operators on two instruments) was averaged to generate the measured concentration in IU/mL. Nominal concentrations of the further serial dilutions in the ZIKV NAT panel were calculated based on the dilution factor.

### Identification of candidate assays and manufacturers

Manufacturers of NAT assays that received FDA EUA approvals for use in blood donor or diagnostic settings were initially invited to participate in a comparative evaluation to establish analytical sensitivity and precise estimation of 50 and 95% LODs of molecular assays for ZIKV.

To expand the initial comparative evaluation study, a call for partners, managed by FIND, was conducted in October 2020 for ZIKV test manufacturers interested in a head-to-head performance evaluation of their assay. Submissions for inclusion in the head-to-head performance evaluation were evaluated based on the following inclusion criteria: 1) the assay must be design-locked and commercially available; 2) the manufacturer must have a quality management system to manufacture tests in place (e.g., ISO certification, good manufacturing practice); and 3) the assay must be approved for use by a regulatory body or have an emergency use authorization. Manufacturers of assays meeting these criteria were invited to be included in the evaluation. Manufacturers who accepted inclusion of their product in the study signed a material transfer agreement to receive panels for the evaluation that included a commitment to allow publication of the study findings.

### Participating labs

**Table 1** summarizes the participating assay manufacturers, testing labs and key characteristics of the assays evaluated in this study. S1 Text provides more detailed summaries of methods and citations for each assay.

### Comparison of diagnostic performance on clinical samples

Residual plasma samples were obtained from individuals with DENV like syndrome or suspected ZIKV virus infection from November 2015 to May 2016 in the states of Tocantins and Alagoas in the North and Northeast regions of Brazil, respectively. To investigate the impact of input sample volume on diagnostic sensitivity, samples were tested for DENV, CHIKV, and ZIKV virus RNA using the research-use Arboplex target-capture transcription-mediated amplification (TC-TMA) assay on the automated Panther system (Grifols Diagnostic Solutions) and the research-use VRI triplex real-time RT-qPCR (rRT-PCR) assay which incorporates the small volume manual RNA extraction method, oligonucleotide sequences and one-step RT-qPCR master mix used for the CDC Trioplex RT-qPCR assay, with amplification performed on a real-time PCR instrument (Roche Lightcycler 480 System). After parallel testing using the Arboplex TC-TMA and VRI triplex RT-qPCR assays, samples with discordant results were re-tested using a primer-probe set and positive control set from the CDC Trioplex RT-qPCR assay (ATCC Item Number NR-50417) on a real-time PCR instrument (Roche Lightcycler 480 System).

### Statistical methods

Probit regression was performed using positive/negative results as the dependent variable and log_10_ concentration (in IU/mL or viral genome equivalents/mL) as the independent variable, with a probit link function. Regression analysis was performed in R (version 3.1.1, R Core Team 2014 and version 4.0.4 R Core Team 2021) using the glm module for generalized linear regression modeling. The fitted probit regression model and its standard errors were used to estimate LOD_50_ and LOD_95_ and confidence intervals. In order to characterize assays’ RNA extraction and amplification efficiency, we computed a novel metric which we term Coefficient of Assay Efficiency (CE). The CE is the product of the LoD in IUs/mL and the derived plasma input in mL, and therefore expresses the number of ZIKV RNA IUs required in the plasma subjected to the RNA extraction and amplification reactions in order to achieve 50% (CE_50_) or 95% (CE_95_) probability of detection.

## Results

### Analytic performance

**Fig 1:**
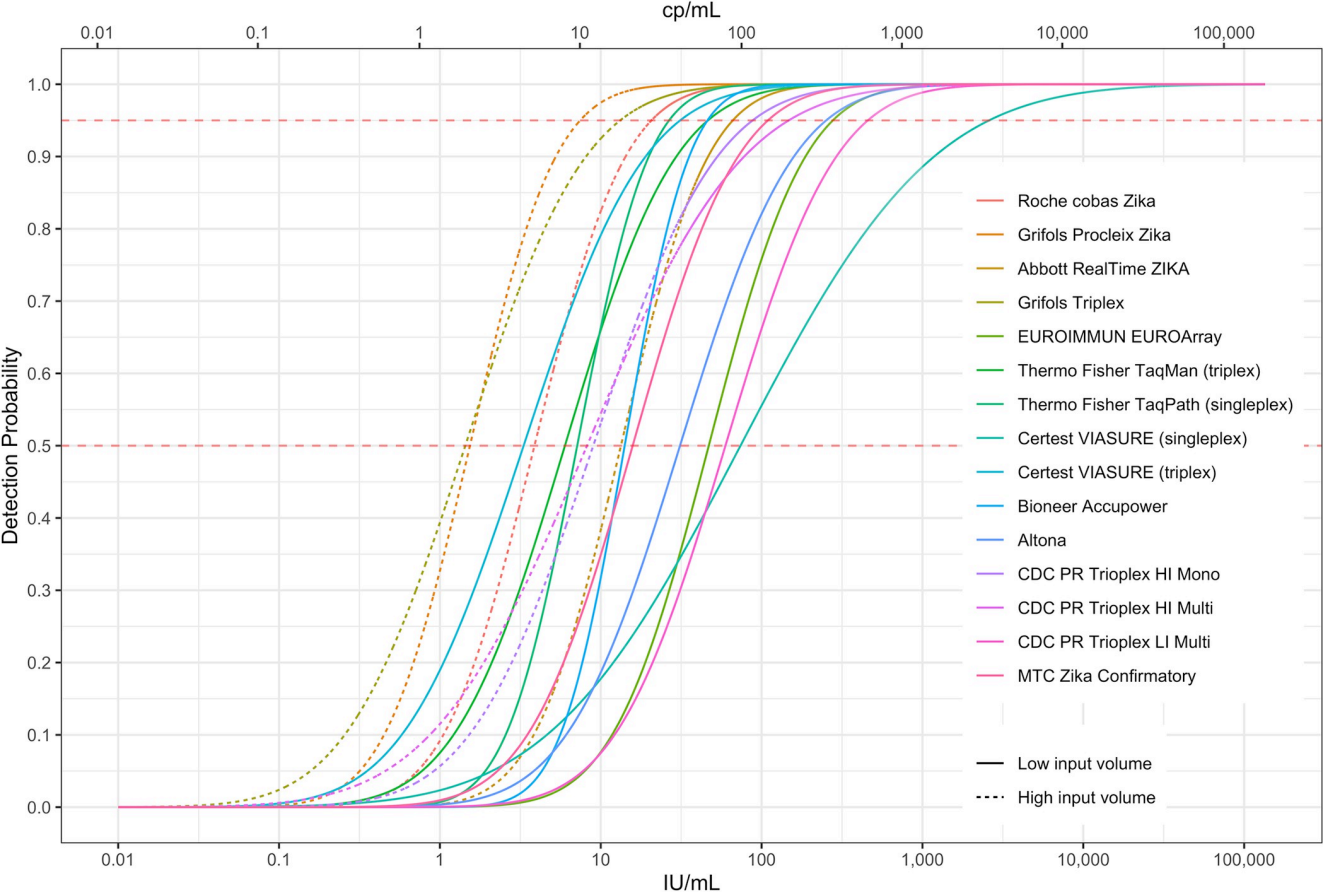
Analytic performance of plasma blood screening and diagnostic assays. Probit curves comparing analytical sensitivity of assays evaluated in this study by replicate testing of Polynesia isolate blinded panels, grouped by assay category or laboratories. The fitted probit regression model curve (solid lines) and standard errors were used to estimate LOD_50_ and LOD_95_ and confidence intervals presented in Table 2.

Results of replicate testing for all 15 individual assays on the unblinded analytical performance panels are reported in **Table 2**, with assays grouped into categories based on similar methods, targets, and findings (LODs). Raw data expressed as number of replicates reactive for each assay are reported in S1 Table. Assays demonstrated good specificity based on analysis of the 25 negative control samples, with only one assay yielding a single reactive result on the negative control. Analytical sensitivities of the assays varied by 1–2 logs based on comparisons of % reactivity on serial dilutions and probit analyses to derive LOD_50_ and LOD_95_, with one outlier that had substantially higher limits of detection than the other evaluated assays (**Fig 1**).

**Table 2:** Probit analysis of 25 replicate serial dilution panel. Results of replicate testing of Polynesia isolate blinded panels presented as combinations of % reactivity and LODs for related assays.

ZIKV RNA c/mL	ZIKV RNA IU/mL	Roche cobas Zika	Grifols Procleix Zika	Abbott RealTime ZIKA	Grifols Triplex	EUROIMMUN EUROArray	Thermo Fisher TaqMan (triplex)	Thermo Fisher TaqPath (singleplex)	Certest VIASURE (singleplex)	Certest VIASURE (multiplex)	Bioneer Accupower	Altona	CDC PR Trioplex HI Mono	CDC PR Trioplex HI Multi	CDC PR Trioplex LI Multi	MTC Zika Confirmatory
2.12E+03	1.58E+03	100%	100%	100%	100%	100%	100%	100%	96%	100%	100%	100%	100%	100%	100%	100%
6.72E+02	5.00E+02	100%	100%	100%	100%	100%	100%	100%	80%	100%	100%	100%	100%	100%	96%	100%
2.12E+02	1.58E+02	100%	100%	100%	100%	92%	100%	100%	72%	100%	100%	84%	100%	100%	84%	100%
6.72E+01	5.00E+01	100%	100%	100%	100%	36%	100%	100%	44%	100%	96%	68%	100%	100%	36%	92%
2.12E+01	1.58E+01	92%	100%	36%	100%	20%	84%	88%	36%	88%	56%	32%	52%	56%	8%	36%
6.72E+00	5.00E+00	52%	88%	20%	76%	4%	28%	20%	4%	48%	8%	8%	24%	24%	8%	8%
2.12E+00	1.58E+00	28%	48%	4%	52%	0%	8%	8%	12%	36%	0%	0%	4%	4%	0%	4%
6.72E-01	5.00E-01	0%	16%	0%	24%	0%	4%	0%	0%	12%	0%	0%	12%	4%	0%	4%
2.12E-01	1.58E-01	0%	0%	0%	0%	0%	4%	0%	0%	0%	0%	0%	0%	0%	0%	0%
6.68E-02	5.00E-02	4%	0%	0%	4%	0%	0%	0%	0%	0%	0%	4%	0%	4%	0%	0%
2.11E-02	1.58E-02	0%	0%	0%	0%	0%	0%	0%	0%	0%	0%	0%	4%	4%	4%	0%
Negative	Negative	0%	0%	0%	0%	4%	0%	0%	0%	0%	0%	0%	0%	0%	0%	0%
**95% LoD (IU/mL)**		**20.7 [11.2,31.9]**	**7.4 [4.1,11.3]**	**66.2 [36.3,101]**	**13.2 [6.5,22.1]**	**277.4 [147.1,435.1]**	**46.4 [23.5,75.9]**	**26.6 [15.5,38.6]**	**1931 [686,4193]**	**31.2 [15.3,52.4]**	**46.1 [27.7,65.2]**	**252 [126,415]**	**87.9 [42.7,149]**	**147 [65.2,271]**	**457 [231,749]**	**109 [56.4,176]**
**50% LoD (IU/mL)**		**3.9 [2.8,5.3]**	**1.5 [1.1,2.1]**	**13.3 [9.8,18.2]**	**1.4 [1,2.1]**	**47.0 [33.9,65.1]**	**6.0 [4.2,8.5]**	**7.1 [5.4,9.5]**	**55.0 [34.5,89.8]**	**3.3 [2.3,4.8]**	**14.1 [10.8,18.4]**	**31.1. [21.8,44.3]**	**9 [6.2,13]**	**8.2 [5.4,12.5]**	**59.6 [42,84.6]**	**15.8 [11.2,22.2]**
**95% LoD (c/mL)**		**27.8 [15.0, 42.9]**	**10 [5.5,15.2]**	**65.8 [42.5,87.8]**	**17.8 [8.7,29.8]**	**372.9 [197.8,584.9]**	**62.4 [31.5,102.0]**	**35.8 [20.8,51.8]**	**2595 [922,5637]**	**41.9 [20.5,70.5]**	**62.0 [37.2,87.6]**	**339.0 [169.8,558.6]**	**118.3 [57.5,200.1]**	**198.6 [87.8,365.6]**	**615.1 [310.1,1007.0]**	**147.2 [75.9,236.7]**
**50% LoD (c/mL)**		**5.2 [3.8,7.2]**	**2.1. [1.5,2.8]**	**27.1 [21.5,34.2]**	**1.9 [1.3,2.8]**	**63.1 [45.5,87.5]**	**8.1 [5.7,11.4]**	**9.6 [7.2,12.7]**	**73.9 [46.4,120.7]**	**4.5 [3.1,6.4]**	**18.9 [14.5,24.8]**	**41.8 [29.3,59.6]**	**12.1 [8.3,17.5]**	**11.0 [7.3,16.8]**	**80.1 [56.6,113.8]**	**21.2 [15.1,29.9]**
**IUs required for 50% detection probability** *		**2.0**	**0.8**	**6.7**	**0.7**	**0.9**	**0.9**	**1.1**	**2.2**	**0.1**	**1.8**	**0.9**	**0.9**	**0.8**	**1.2**	**1.7**
**IUs required for 95% detection probability** *		**10.4**	**3.7**	**33.1**	**6.6**	**5.5**	**7.0**	**4.0**	**77.2**	**1.2**	**5.8**	**7.1**	**8.8**	**14.7**	**9.1**	**11.6**

* IUs required for detection in the derived plasma input (coefficient of assay efficiency, CE).

c/ml = copies of viral genome equivalents per milliliter

The Roche and Grifols blood screening qualitative NAT assays had similar single viral genome equivalents sensitivities at LOD_50_. The Grifols assay had somewhat higher analytic sensitivity based on a LOD_95_ of 7.4 IU/mL (95% CI: 4.1,11.3) compared to 20.7 IU/mL (95% CI: 11.2,31.9) for the Roche assay.

Diagnostic PCR assays performed similarly with analytical sensitivities in the range of 6.0 to 55.0 IU/ml at the 50% probability of detection level. The Certest singleplex assay had a substantially higher LOD_95_ than the other assays at 1,931 IU/mL. The 95% limits of detection of the Altona and MTC lower input volume assays were 252 IU/ml (95% CI: 126,415) and 109 IU/mL (95% CI: 56,176), respectively.

The analytical sensitivity for detection of ZIKV RNA of the Grifols singleplex assay was similar to that of the Grifols multiplex ZIKV/DENV/CHIKV assay, with LOD_95_ of 7.4 IU/mL (95% CI: 4.1,11.3) and 13.2 IU/mL (95% CI: 6.5,22.1), respectively, which was not statistically significant. The ThermoFisher singleplex assay (LOD_95_ 26.6 IU/mL, 95% CI: 15.5,38.6) also had similar sensitivity to the ThermoFisher multiplex assay (LOD_95_ 46.4 IU/mL, 95% CI: 23.5,75.9). By contrast, the Certest singleplex assay had substantially poorer sensitivity (LOD_95_ 1,931 IU/mL, 95% CI: 686,4,193) than the Certest arbovirus multiplex assay (LOD_95_ 31.2 IU/mL, 95% CI: 15.3,52.4), owing to optimizations made to the multiplex assay that had not been incorporated in the ZIKV assay (personal communication from Certest).

CE_50_ values ranged from 0.13 for the Certest VIASURE multiplex and 0.75 for the Grifols multiplex assays, indicating very high RNA extraction and amplification efficiency, to 6.65 for the Abbott RealTime assay. In general assays using transcription mediated amplification (TMA) technology had greater efficiency than assays using conventional PCR technology for ZIKV RNA detection. Some assays, like the CDC Trioplex, with very good efficiency nevertheless had relatively poor analytical sensitivity as a result of low effective input volumes.

### Impact of diagnostic assay performance

To investigate the impact of input sample volume on diagnostic sensitivity of two of the multiplex assays detecting DENV, CHIKV and ZIKV RNA on clinical specimens, we tested 1017 plasma samples collected from individuals with dengue like syndrome or suspected ZIKV infection in early 2016 in Brazil. Samples were tested using the Grifols Arboplex TC-TMA assay and the VRI small volume triplex RT-qPCR assay. Samples with discordant results between the two assays were further tested using the small volume manual extraction protocol and oligonucleotides from the CDC Trioplex RT-qPCR assay on the Roche Lightcycler 480 instrument. Both the TC-TMA and RT-qPCR assays detected DENV RNA in 2% (21/1017), CHIKV RNA in 19% (189/1017) and ZIKV RNA in 7% (72/1017) of the plasma specimens. Rare (0.1%) coinfections were also detected by both assays (1/1017 each for DENV-CHIKV, DENV-ZIKV and CHIKV-ZIKV). In addition, the TC-TMA assay detected DENV RNA in 1.7% (17/1017), CHIKV RNA in 4.3% (44/1017) and ZIKV RNA in 4.6% (47/1017) of the plasma specimens that were not detected by rRT-PCR. Furthermore, the TC-TMA assay detected 0.2% (2/1017) DENV-CHIKV and 0.4% (4/1017) CHIKV-ZIKV coinfections that were not detected by rRT-PCR, as well as 0.2% (2/1017) CHIKV-ZIKV and 0.1% (1/1017) DENV-ZIKV coinfections that were only detected as ZIKV infections by rRT-PCR. In no cases was viral RNA detected by RT-qPCR but not by TC-TMA. Overall, the data showed that testing by TC-TMA had increased diagnostic sensitivity relative to testing by RT-qPCR of 47% for DENV, 21% for CHIKV and 40% for ZIKV.

## Discussion

Appropriate diagnostic preparedness includes rapid deployment of validated assays with optimized performance characteristics. This study used blinded replicate testing of serial dilutions of well characterized ZIKV viral stocks to robustly characterize the relative analytical sensitivities of ZIKV molecular assays used for clinical diagnosis, research and blood donor screening. When performed according to manufacturer instructions for use (IFU), assays demonstrated consistently high specificity, however differences in sensitivity were noted.

Enhancing sensitivities of diagnostic assays may be achievable by increasing sample input per assay, as demonstrated by the relative sensitivities of CDC LI and HI assays performed at the two participating CDC laboratories. Donor screening NAT assays and a Grifols research use Triplex (ZIKV/DENV/CHIKV) assay were comparable in sensitivity and significantly more sensitive than diagnostic RT-qPCR assays, due to higher input volumes of plasma. There were substantial differences in volumes of plasma subjected to amplification in the blood screening versus diagnostic assays. More impactful in performance the volume of plasma subjected to extraction, which correlated with analytical sensitivity demonstrating that enhanced sensitivity was associated with using a greater sample volume of plasma input for initial RNA extraction. The significantly poorer sensitivities of diagnostic PCR assays are primarily attributable to the lower volumes of plasma extracted and consequently of eluted RNA amplified by different assays. Ideal assays to maximize sensitivity and throughput may not be a viable option in all contexts and other factors such as cost, instrumentation and regulatory approval status may influence assay availability and selection, particularly in resource constrained settings.

Although the input volumes of plasma processed in different assays is a primary driver of sensitivity, RNA extraction and amplification reaction efficiency also contribute to assay performance. The coefficient of efficiency (CE_50_) is a measure of input target viral genome equivalents number as a ratio of the 50% LOD relative to the volume of plasma subjected to amplification. The Grifols TMA assays demonstrated very high CE_50_ performance, likely due to the efficiency of single tube target capture RNA concentration and the isothermal amplification step. Conventional RT-qPCR assays had somewhat lower and variable CE50 performance, which could be impacted by both extraction and application efficiency. The CDC Trioplex had relatively poor analytical sensitivity despite high CE_50_, highlighting the importance of input volumes in driving assay sensitivity.

Expanded multiplexed arbovirus assays are needed for clinical diagnostics, blood screening and surveillance, particularly in areas where multiple arboviruses co-circulate. However differential sensitivities of diagnostic assays have implications for diagnosis and management of clinical cases and monitoring of travelers and pregnant women. This is particularly significant in arbovirus endemic settings where in the absence of laboratory confirmation, clinical diagnosis is challenged because of similar clinical presentation of infection with ZIKV, DENV, CHIKV and other regionally endemic infections; specific identification of ZIKV infections is particularly critical for prenatal and postnatal counseling and clinical management. [30,31].

The higher sensitivity and CE_50_ performance observed on the ZIKV molecular panel for high volume plasma input multiplexed TMA assay relative to low volume plasma input multiplexed RT-qPCR assay was corroborated on clinical samples, with 40% increased diagnostic sensitivity for ZIKV using high effective input volume TMA compared to low effective input volume RT-PCR. Likewise, the diagnostic sensitivity for DENV and CHIKV were 47% and 21% higher, respectively, using high input TMA relative to low input RT-PCR. This data supports the use of high input multiplexed TMA as a sensitive test for clinical screening of plasma in geographic areas with arbovirus outbreaks that may include co-circulation of ZIKV, DENV and CHIKV.

Some limitations of this evaluation exist. The molecular panels did not include clinical specimens and are thus limited to determination of limit of detection of a single ZIKV strain. Panels based on a single viral isolate do not represent all epidemiologic scenarios or diverse global geographic areas where ZIKV is known to occur or where ZIKV transmission may emerge or re-emerge (e.g., Africa, southeast Asia). The performance of assays on different ZIKV variants is not well understood, and although the target region of many commercial assays is propriety, assays are generally designed to target conserved regions to enable detection across variants. This study was executed under optimal conditions as assays were performed by the manufacturers and hence the findings may not translate directly to real world performance. Ideally, it is the combination of rigorous assay evaluation and execution of laboratory proficiency programs that will ensure the most reliable diagnostic interpretation; in this context ongoing External Quality Assurance (EQA) and proficiency testing should be considered [32]. Although this evaluation focuses on high throughput, commercially available assays, it should be noted that well-established in-house protocols may also perform well. Finally, this panel focuses on assay performance using serum, comparative evaluation of assays using alternative sample types for use in different diagnostic contexts should be considered.

Opportunities exist for additional external evaluations of commercialised ZIKV tests as remaining panels from the current study exist that can be used in the future for analytical evaluation of commercialised molecular assays used by laboratories for ZIKV diagnosis but for which the test developers did not respond to FIND’s call for partners. Based on this ongoing work, WHO is updating priority use cases and target product profiles for diagnostics. This should include consideration of contexts for recommendations, sample acquisition, and selection of assays, e.g., sample acquisition linked to blood donor screening assays where ample volumes may be available, versus clinical samples with lower volumes, and less resourced areas with more rudimentary molecular capabilities. Additionally, these panels could be expanded to include samples from other epidemiologically diverse endemic regions (sub-Saharan Africa, south-east assaysoutheast Asia). Outcomes of this evaluation will serve to accelerate knowledge on test performance and inform public health policy, surveillance programs, blood donor screening and clinical management of patients with suspected ZIKV infection.

## Supporting information

S1 Table Probit analysis of 25 replicate serial dilution panel. Results of replicate testing of Polynesia isolate blinded panels presented as number of replicates reactive for related assays.
(XLSX)

S1 Text Supplemental methods. (DOCX)
