# Supplementary material for: Standardized evaluation of Zika nucleic acid tests used in clinical settings and blood screening
Source: PLoS Negl Trop Dis. 2023 Mar 17;17(3):e0011157. doi: 10.1371/journal.pntd.0011157 (PMC10072466; doi:10.1371/journal.pntd.0011157)
Supplement: S1 Text — (DOCX) [file pntd.0011157.s002.docx]

*S1. Supplemental Methods*

*Roche*

The cobas Zika test has been specifically designed utilizing the generic cobas omni Utility Channel on the cobas 6800/8800 Systems. Samples were tested with input volume of .5ml. The cobas Zika test is based on fully automated sample preparation (nucleic acid extraction and purification) followed by PCR amplification and detection. The cobas 6800/8800 Systems consist of the sample supply module, the transfer module, the processing module and the analytic module. Data management is automated and performed by the cobas 6800/8800 software which assigns test results for all tests as non-reactive, reactive, or invalid.

*Hologic*

Procleix Zika Virus assay is a qualitative in vitro assay to detect Zika RNA on a fully automated Panther system. The assay is based on the same technology as other Procleix assays, which involve three main steps: sample preparation using magnetic based target capture, RNA amplification by transcription-mediated amplification (TMA), and detection of the amplification products by the hybridization protection assay (HPA) using chemiluminescent probes. To mitigate the risk of false negative results, the assay targets 2 separate regions of the ZIKV genome and includes an internal control to validate each reaction. The assay uses 0.5mL input volume and all steps in one tube with an internal control (IC) incorporated. The IC is added into samples via the working target capture reagent, to monitor target capture, amplification, and detection, as well as operator or instrument error. The analyte and IC probes are labeled by chemiluminescent and have different light emission kinetics, which are distinguishable from one another. Results are expressed in relative light units (RLU) values and signal-to-cutoff (S/CO) values. A floating cutoff value is obtained with calibrators included in each run. A sample is considered “Reactive” if the analyte S/CO is great than or equal to 1.0. A sample is considered “Nonreactive” if the analyte S/CO is less than 1.0 and the internal control signal is greater than the internal control cutoff. The sample result is considered “invalid” if the internal control signal is greater than the set maximum, or both the analyte S/CO is less than 1.0 and the internal control signal is below the internal control cutoff. The sample with invalid result must be retested. Analytical sensitivity was determined by probit analysis of results from testing 20-72 replicates per level of serially diluted in vitro synthesized RNA transcript and a ZIKV positive plasma specimen that was quantified by real-time PCR and digital PCR. Specificity was assessed by testing 675 plasma blood donor samples collected from a non-endemic region in the US.

*CDC Puerto Rico*

*CDC Singleplex EUA*

RT-PCR was performed as previously described [31]. Briefly, two real-time primer/probe sets specific for the ZIKV 2007 strain were used with 5-FAM labeled reporter probe). All real-time assays were performed using the QuantiTect Probe RT-PCR Kit (QIAGEN, Valencia, CA, USA) and amplified in an iCycler instrument (Bio-Rad, Hercules, CA, USA) according to the manufacturer’s protocol. Specificity of the ZIKV primers was extensively evaluated yielding negative results. Sensitivity was evaluated by testing dilutions of known copy numbers of an RNA transcript of the ZIKV 2007 sequence. RNA was quantified using the Ribogreen RNA-specific Quantitation Kit (Invitrogen) and the TBE-380 mini-fluorometer (Turner Biosystems, Sunnyvale, CA, USA).

*CDC PR Trioplex EUA*

The trioplex assay is used for the simultaneous and qualitative detection and differentiation of dengue, chikungunya and Zika Viruses from a single sample. Extraction of 1ml was performed on MagNA Pure LC Total Nucleic Acid Isolation Kit (Roche) and eluted in 100ul. PCR reactions were amplified with 10ul extracted RNA using the SuperScript III Platinum One -Step qRT -PCR Kit (ThermoFisher) on 10 replicates using an Applied Biosystems 7500 Fast Dx Real-Time PCR Instrument (ThermoFisher Scientific)

VRI Zika Alternate NAT assays

Zika alternate NAT test was performed on donor plasma or RBC samples using manual nucleic acid extraction (QIAamp Viral RNA Mini Kit, Qiagen Inc., Germantown, MD) with 140 µL specimen input volume and 60 µL elution volume. For RBC samples, extraction was modified from the manufacturer’s instructions with the addition of a second wash with each of the two wash buffers. cDNA synthesis immediately followed by 45 cycles of PCR amplification (SuperScript III Platinum One-Step qRT-PCR Kit, Thermo Fisher Scientific, Waltham, MA) were performed with Zika primer/probe sequences developed by the CDC^1,2^ in duplicate 50-µL reactions each containing 22.7 µL RNA on a real-time instrument (LightCycler 480 System, Roche Diagnostics Corporation, Indianapolis, IN). Results were interpreted as equivocal or positive if one or both duplicate reactions generated a fluorescence curve that crossed the threshold within 40 cycles, respectively. Estimated viral loads were calculated relative to a standard curve created by serially diluting Zika virus (strain PF13/251013-18) with known copy number in plasma and extracting and processing with each batch of donor samples. The 50% and 95% limit of detection for this assay are 42.6 and 347 copies/mL, respectively.^3^

^1^Lanciotti RS, Kosoy OL, Laven JJ, et al. Genetic and serologic properties of Zika virus associated with an epidemic, Yap State, Micronesia, 2007. Emerg Infect Dis 2008;14:1232-9.

^2^Trioplex Real-time RT-PCR Assay [Internet]. Silver Spring (MD): U.S. Food and Drug Administration; last updated 2016 Sep 21 [cited 2016 Nov 23]. Available from: http://www.fda.gov/downloads/MedicalDevices/Safety/EmergencySituations/UCM491592.pdf

^3^Bakkour S, Lee T, Stone M, et al. Analytical performance of PCR assays for detection of Zika virus RNA [abstract]. Transfusion 2016;56(Suppl):202A.
